# Transcription and chromatin determinants of de novo DNA methylation timing in oocytes

**DOI:** 10.1186/s13072-017-0133-5

**Published:** 2017-05-12

**Authors:** Lenka Gahurova, Shin-ichi Tomizawa, Sébastien A. Smallwood, Kathleen R. Stewart-Morgan, Heba Saadeh, Jeesun Kim, Simon R. Andrews, Taiping Chen, Gavin Kelsey

**Affiliations:** 1 Epigenetics Programme, Babraham Institute, Cambridge, CB22 3AT UK; 2 Department of Histology and Cell Biology, School of Medicine, Yokohama City University, Yokohama, 236-0004 Japan; 3 Department of Epigenetics and Molecular Carcinogenesis, The University of Texas M.D. Anderson Cancer Center, Smithville, TX 77030 USA; 4 Bioinformatics Group, Babraham Institute, Cambridge, CB22 3AT UK; 5 Centre for Trophoblast Research, University of Cambridge, Cambridge, CB2 3EG UK; 6 Laboratory of Developmental Biology and Genetics, Department of Molecular Biology, University of South Bohemia, 37005 Ceske Budejovice, Czech Republic; 7 Friedrich Miescher Institute for Biomedical Research, 4058 Basel, Switzerland; 8 Biotech Research and Innovation Centre (BRIC), University of Copenhagen, 2200 Copenhagen, Denmark; 9 Computer Science Department, KASIT, University of Jordan, Amman, Jordan

**Keywords:** Oocytes, DNA methylation, Histone modifications, Transcription, Imprinting

## Abstract

**Background:**

Gametogenesis in mammals entails profound re-patterning of the epigenome. In the female germline, DNA methylation is acquired late in oogenesis from an essentially unmethylated baseline and is established largely as a consequence of transcription events. Molecular and functional studies have shown that imprinted genes become methylated at different times during oocyte growth; however, little is known about the kinetics of methylation gain genome wide and the reasons for asynchrony in methylation at imprinted loci.

**Results:**

Given the predominant role of transcription, we sought to investigate whether transcription timing is rate limiting for de novo methylation and determines the asynchrony of methylation events. Therefore, we generated genome-wide methylation and transcriptome maps of size-selected, growing oocytes to capture the onset and progression of methylation. We find that most sequence elements, including most classes of transposable elements, acquire methylation at similar rates overall. However, methylation of CpG islands (CGIs) is delayed compared with the genome average and there are reproducible differences amongst CGIs in onset of methylation. Although more highly transcribed genes acquire methylation earlier, the major transitions in the oocyte transcriptome occur well before the de novo methylation phase, indicating that transcription is generally not rate limiting in conferring permissiveness to DNA methylation. Instead, CGI methylation timing negatively correlates with enrichment for histone 3 lysine 4 (H3K4) methylation and dependence on the H3K4 demethylases KDM1A and KDM1B, implicating chromatin remodelling as a major determinant of methylation timing. We also identified differential enrichment of transcription factor binding motifs in CGIs acquiring methylation early or late in oocyte growth. By combining these parameters into multiple regression models, we were able to account for about a fifth of the variation in methylation timing of CGIs. Finally, we show that establishment of non-CpG methylation, which is prevalent in fully grown oocytes, and methylation over non-transcribed regions, are later events in oogenesis.

**Conclusions:**

These results do not support a major role for transcriptional transitions in the time of onset of DNA methylation in the oocyte, but suggest a model in which sequences least dependent on chromatin remodelling are the earliest to become permissive for methylation.

**Electronic supplementary material:**

The online version of this article (doi:10.1186/s13072-017-0133-5) contains supplementary material, which is available to authorized users.

## Background

The establishment of DNA methylation in the female germline in mammals is essential for genomic imprinting and successful development of the embryo following fertilisation [1–3]. Following genome-wide erasure of methylation in primordial germ cells [4], mammalian oocytes acquire a highly structured DNA methylation landscape in which domains of uniform methylation are separated by extensive unmethylated domains [5, 6]; this largely bimodal pattern is unique amongst mammalian cell types. DNA methylation is associated mostly with transcriptionally active gene bodies in oocytes, and these methylated domains contain intragenically located CpG islands (CGIs) that also gain methylation, including the germline differentially methylated regions (gDMRs) of imprinted genes [5–7]. As a result, there is highly programmed methylation of a defined set of ~2000 CGIs in oocytes, mostly on account of their location within active transcription units. We, and others, have shown that transcription is functionally required to define methylation in oocytes: Abrogating specific transcription events prevents methylation of the associated loci, including at imprinted gDMRs [6, 8, 9].

The oocyte represents a pure de novo methylation system, as an entire DNA methylation landscape is established on an essentially unmethylated genome in a non-dividing cell [10]; therefore, it provides a unique opportunity to investigate the extent to which different sequence features acquire methylation as a result of common or distinct mechanisms. Current knowledge is largely limited to the fully established DNA methylome in fully grown oocytes at the germinal vesicle (GV) stage or in ovulated metaphase II (MII) oocytes [5, 11], such that differences in the mechanistic requirements for methylation of various sequence elements or in the kinetics of their methylation are obscured. Thus, investigating methylation at intermediate stages would be informative, but genome-wide studies have not yet been done. Analysis of a limited number of imprinted gDMRs identified that de novo methylation is a function of developmental stage of follicles and oocyte size, with methylation initiated around the time follicles transition into the antral or secondary follicle stage of development. Moreover, locus-specific analysis has shown that the onset and progression of methylation appear to differ between imprinted gDMRs [12–14]. This asynchrony has functional importance, as nuclear transfer experiments have shown that different imprinted domains acquire imprinting competence at different stages of oocyte growth [15].

In view of the rather simple methylation landscape of the oocyte, the differential timing of methylation acquisition at gDMRs is unexpected, and the reasons for this asynchrony are unclear. Understanding its basis is essential for identifying the origin of methylation defects in oocytes that could underlie some errors in imprinting. Such asynchrony also suggests that different factors, or combinations of factors, may be necessary for methylation of different gDMRs, individual CGIs or individual methylated domains, aside from the common requirement for the de novo DNA methyltransferase DNMT3A and its obligate partner DNMT3L [5, 7, 11]. Given the strong association with transcription [6], and major changes in the transcription programme during oocyte growth [16], one possibility is that the timings of transcription events traversing gDMRs and CGIs could account for differences in the onset of methylation at individual elements.

At a mechanistic level, de novo DNA methylation occurs in a chromatin template and, in accordance with the biochemical properties of DNMT3A and DNMT3L [17–19], is predicted to depend upon the acquisition of a permissive histone modification state. Thus, regions destined for DNA methylation are proposed to be marked by histone 3 trimethylated at lysine 36 (H3K36me3) and should lack H3 di- or trimethylated at lysine 4 (H3K4me2/me3) [7, 20]. Evidence in support of this model is the requirement for the H3K4 demethylase KDM1B for DNA methylation of most imprinted gDMRs and CGIs that acquire methylation in oocytes and the increase in H3K36me3 at these elements during oocyte growth [20, 21]. Such chromatin state changes may also be downstream of transcription events: H3K36me3 is deposited by SETD2 in association with elongating RNA polymerase II [22–24], although the role of SETD2 in oocytes has not yet been determined; and removal of H3K4me2 and gain of H3K36me3 at the gDMR of the imprinted locus *Zac1* in oocytes was shown to depend on transcription from an upstream, oocyte-specific promoter [6].

To investigate how transcription influences the kinetics of methylation at gDMRs and throughout the genome, we generated genome-wide DNA methylation and high-resolution transcriptome maps of size-selected populations of growing oocytes spanning the onset of methylation. We find that the major remodelling of the oocyte transcriptome occurs well before the onset of DNA methylation, indicating that initiation of transcription events is not temporally coupled to methylation of specific loci. However, rate of gene body methylation does correlate with transcription level, which could reflect the degree of transcription-coupled chromatin remodelling. CGI methylation timing reflects (1) the H3K4me2 levels found in non-growing and early growing oocytes, (2) dependence on H3K4 demethylases and (3) presence of specific transcription factor motifs, supporting a model in which sequences requiring less chromatin remodelling are the earliest to become permissive for de novo methylation.

## Results

### Capturing the onset of de novo DNA methylation in oocytes

To analyse the onset and progression of de novo methylation at a genome-wide scale, we isolated growing oocytes from pre-pubertal mouse ovaries (post-natal days 7–18) and sorted them into the following, non-overlapping size categories: 40–45, 50–55 and 60–65 μm. Genome-wide methylation maps were generated by bisulphite conversion of oocyte DNA and Illumina sequencing. For unbiased genome coverage to enable interrogation of all sequence features in 60–65 μm oocytes, we applied post-bisulphite adapter tagging (PBAT; [25]); for focussed coverage of CGIs and other GC-rich sequences in all three size classes of oocytes, we applied reduced representation bisulphite sequencing (RRBS; [7]). The 60–65 μm PBAT library yielded 98,951,299 uniquely mapped read pairs, covering 18,651,142 (85.3%) of mappable CpG sites at ≥1 read and 5,731,851 CpGs (26.3%) with ≥5 reads. The RRBS libraries covered between 551,677 and 838,372 CpG sites (≥5 reads) and 13,944–15,799 (60.6–68.7%) of the 23,009 CGIs in the mouse autosomes and X chromosome (CGI coverage threshold ≥5 CpG sites with ≥5 reads; Additional file 1: Table S1). The PBAT and RRBS data were compared with published data sets from non-growing oocytes (NGO) and GV or MII oocytes [5, 7, 11]. In parallel, RNA sequencing (RNA-seq) libraries were made from similar pools of size-selected oocytes (see below).

**Fig. 1:**
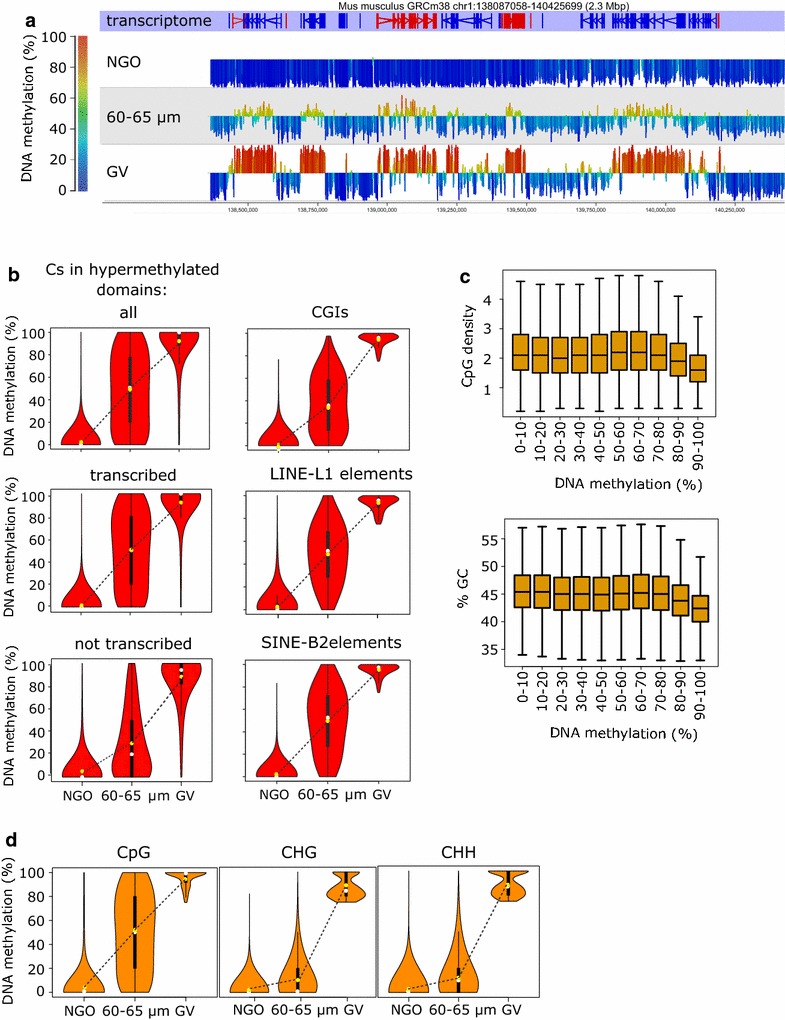
Rates of de novo DNA methylation of different sequence features in growing oocytes. **a** Screenshot of a 2.3-Mb interval of chromosome 1 depicting methylation in NGOs, 60–65 µm and GV oocytes. *Vertical bars* represent mean methylation of 2-kb windows, with 1-kb steps, *height and colour* denoting % methylation. The *horizontal lines* are set at 50% methylation, with higher levels of methylation *above the line* and lower levels *below the line* and *shaded* according to the *colour scale on the left*. The 60–65 µm data are from PBAT from the current manuscript; NGO and GV data are from [5, 11]. **b** *Violin plots* showing distribution of CpG methylation values in all hypermethylated domains, transcribed hypermethylated domains (≥90% of the length of the domain covered by transcript, domains ≥5 kb), transcriptionally silent hypermethylated domains (≤10% of the length of the domain covered by transcript, domains ≥5 kb), CGIs, LINE L1s and SINE-B2s in NGO, 60–65 µm and GV oocytes. *Shape of the violin plot* represents Kernel density estimation, i.e. probability density of the data at the different values. *White dots* correspond to the median, *yellow dots* to the average, *bold lines* the interquartile range and thin lines adjacent values, i.e. minimum and maximum values within the ×1.5 interquartile range from the first and third quartile, respectively. **c** *Box whisker plots* reporting CpG density and GC content of 2-kb genomic regions that are fully methylated in GV oocytes (≥75% DNA methylation) categorised according to their % DNA methylation in 60–65 µm oocytes (*x* axis). *Boxes*, interquartile range, with bar as median and *whiskers* as ×1.5 interquartile range, outliers not shown. Between 3619 and 30320 2-kb intervals were analysed in each methylation category. **d** *Violin plots* showing methylation levels of Cs in CpG, CHG and CHH contexts in NGOs and 60–65 µm oocytes of Cs that are fully methylated (≥75%) in GV oocytes

The overall CpG methylation level of 60–65 μm oocytes determined by PBAT was 22.25%, compared with 2.36% in NGOs and 38.68% in GV oocytes, showing that this stage represents a midpoint in the progression of global de novo methylation (Fig. 1a; Additional file 2: Table S2). We then evaluated whether all genomic features that become methylated in GV oocytes gain methylation at similar rates, including the hypermethylated domains of GV oocytes we previously designated [6]. CpGs in hypermethylated domains have attained on average 48.00 ± 0.02% methylation in 60–65 μm oocytes (Additional file 2: Table S2), although there is a considerable spread in the methylation level of these CpGs at this time (Fig. 1b). We previously showed that 85–90% of hypermethylated domains were associated with transcription units active in oocytes [6]; therefore, we asked whether domains associated with transcription units and those apparently not associated with transcription displayed similar kinetics of methylation. Comparison of CpG methylation rate of transcribed hypermethylated domains and apparently transcriptionally silent hypermethylated domains revealed that CpGs in transcriptionally silent regions are methylated later: average CpG methylation in transcribed domains is 50.1% but 30.0% for transcriptionally silent regions (Fig. 1b). For CGIs that become methylated fully (≥75%) in GV oocytes, mean methylation (37.21 ± 0.69%) in 60–65 μm oocytes was less than most other sequence features (Fig. 1b; Additional file 2: Table S2). An effect of CpG density is also apparent when considering 2-kb genomic windows: regions of highest methylation (≥80%) in 60–65 μm oocytes had on average lower CpG density and GC content (Fig. 1c).

Similar to hypermethylated domains, most classes of transposable element (TEs) that become methylated (≥75%) in GV oocytes are midway in methylation progression (Fig. 1b; Additional file 2: Table S2, Additional file 3: Fig. S1A), although there was interesting variation in the kinetics of specific elements. Some TEs start at a higher level of methylation in NGOs, such as some endogenous retroviral (ERVK) long-terminal repeat (LTR) elements, reflecting incomplete erasure of methylation in primordial germ cells [4]. In addition, there was a significant variation in the rate of methylation of specific TE subfamilies. Notably, of the 20 most abundant LINE-L1 subfamilies, methylation of three of the four L1Md subfamilies was significantly delayed (average methylation of L1Md_A 39.9%, L1Md_F3 44.3% and L1Md_T 42.0%, compared with 48.1–54.4% for the remaining L1 subfamilies). In comparison, there were no differences in the methylation rate of the 20 MaLR subfamilies (Additional file 3: Fig. S1B). L1Md elements are amongst the youngest L1s, with the least degenerated sequence, the most intact transcription factor (TF) binding sites and which have to be actively suppressed [26, 27]. Many of the L1Md subfamilies also retained residual methylation in NGOs (6.5–19.9%, compared with 1.4–3.7% for other L1s). These results indicate that different sequence features acquire methylation with similar overall kinetics, suggesting that the de novo methylation complex is not targeted preferentially to any particular sequence feature. However, the delayed methylation of CGIs and specific L1 subfamilies, as well as at untranscribed regions, points to additional or alternative mechanistic requirements at these elements.

**Fig. 2:**
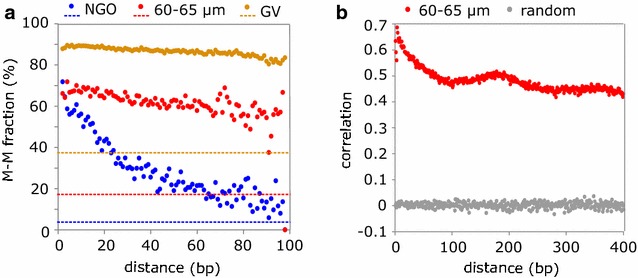
Properties of ongoing de novo DNA methylation in growing oocytes. **a** Average proportion of neighbouring CpG pairs where both CpGs are methylated (M–M fraction) by distance of CpG pairs in NGO, 60–65 µm and GV oocytes. The value of the M–M fraction was quantified for each possible distance between two neighbouring CpGs on the same sequencing read using formula M–M pairs/(M–M + M–U pairs), where M–U pairs represent CpG pairs where upstream CpG is methylated and downstream unmethylated. Only reads mapping to chromosome 1 were analysed. The *horizontal lines* represent the genomic average methylation level of each stage. **b** The distant-dependent correlation of methylation between CpG pairs in 60–65 µm oocytes, compared with random-shuffled data

In fully grown oocytes, there is a high level of concordance in methylation of adjacent CpGs across the extensive hypermethylated domains [6]. Having captured oocytes midway in the progression of methylation, we looked at the coherence of ongoing methylation to investigate co-operativity of the de novo methylation complex. For each sequencing read containing multiple CpGs, we asked how often and over what distance CpGs had the same methylation state. For 60–65 μm oocytes, neighbouring CpGs were both methylated 60–70% of the time over 60 bp and at least 50% of the time over 90 bp (Fig. 2a). If CpG sites were being methylated individually without co-operativity, the probability that CpG pairs were both methylated would equate to the overall genomic methylation level which, in 60–65 μm oocytes, was 17.56%. Therefore, these data indicate co-operativity in methylation of adjacent CpGs by DNMT3A/DNMT3L during oocyte growth, similar to findings of DNMT3B function in embryonic stem cell (ESC) [28]. We note, also, that although concordance of methylation declines with distance, there is a local maximum in the correlation at ~180 bp (Fig. 2b), which approximates the size of a nucleosome, consistent with a model in which de novo methylation occurs in linker regions, as proposed in ESCs [28]. Finally, oocytes have been shown to have extensive methylation outside of the CpG context, in that methylation of non-CpG sites accounts for more than half of the total amount of methylated cytosine [11, 29]. We looked specifically at all informative cytosines that become fully methylated (≥75%) in GV oocytes. Strikingly, in 60–65 μm oocytes, CHG and CHH sites (where H = A, T or C) that become methylated in GV oocytes were only 10.77 ± 0.08 and 13.90 ± 0.05% methylated, respectively, compared with 49.31 ± 0.05% for CpG sites, indicating preferential methylation of CpG sites during oocyte development (Fig. 1d; Additional file 4: Table S3).

### CGIs and imprinted gDMRs gain DNA methylation at different rates in oocytes

**Fig. 3:**
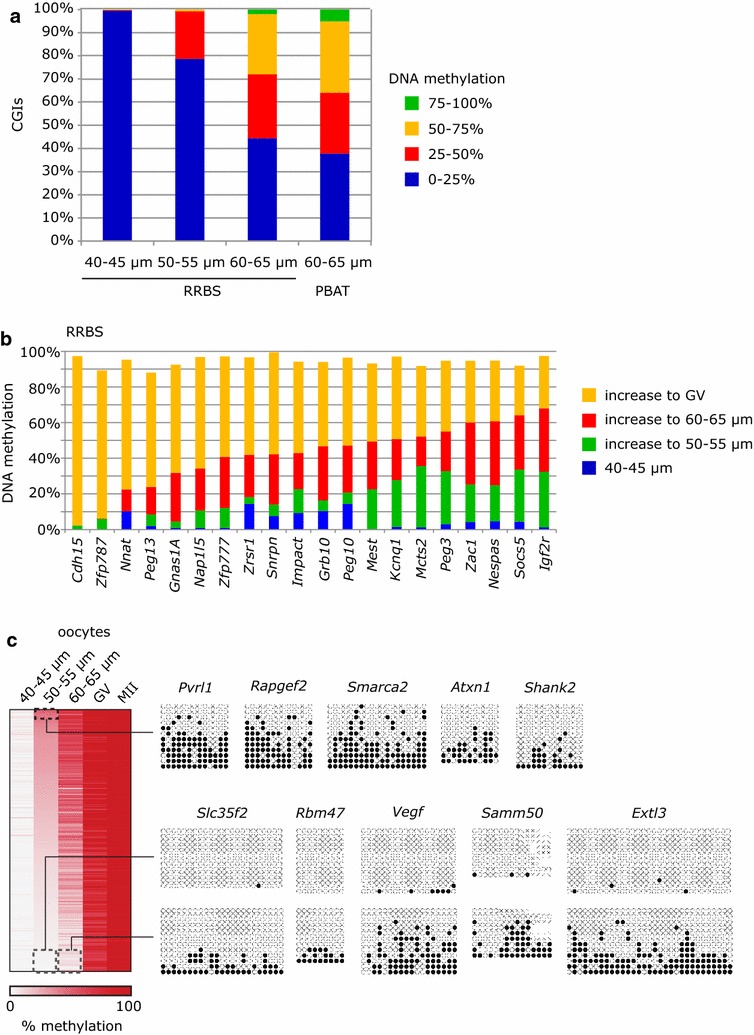
CpG islands gain DNA methylation at different rates in growing oocytes. **a** Barchart of CGI methylation in the oocyte size populations from the RRBS and PBAT datasets. The number of CGIs covered in each dataset is given in Additional file 1: Table S1. **b** Methylation of gDMRs in RRBS datasets, displaying the basal level in 40–45 µm oocytes, and the increases in methylation to the subsequent size populations. gDMRs are ordered according to their methylation level in 60–65 µm oocytes, which is comparable with PBAT data (see Additional file 3: Fig. S2A). **c** Validation of CGI methylation in different oocyte size populations. Heatmap shows methylation progression at CGIs that become methylated between 40 and 45 µm and MII oocytes (data from published GV and MII RRBS datasets). Five early-methylating CGIs and five late-methylating CGIs were selected, and their methylation in 50–55 µm oocytes (early-methylating CGIs) or both 50–55 and 60–65 µm oocytes was confirmed by locus-specific bisulphite sequencing. *White dots* represent unmethylated CpGs and black dots methylated CpGs

To look in more detail at the progression of methylation at CGIs, we considered the RRBS datasets. There is very little methylation of CGIs in 40–45 μm oocytes: only three CGIs (of 522 CGIs with sufficient data) that become fully methylated in GV oocytes were methylated ≥25% in 40–45 μm oocytes, and two of these have residual methylation in NGOs [7]. Methylation was first detected in the 50–55 μm size class (22% of CGIs destined for full methylation having ≥25% methylation in this size group) and at least 55% of CGIs showed intermediate (25–75%) to high (≥75%) levels of methylation in 60–65 μm oocytes (Fig. 3a). Overall, there was a very high level of correlation (*R* = 0.929) between the RRBS and PBAT libraries in CGI methylation at the 60–65 μm stage (Additional file 3: Fig. S2A), suggesting that the differences in level of methylation are reproducible and biological in origin. Focussing on imprinted gDMRs, methylation in 60–65 μm oocytes assessed by the two methods ranged from 0 to ~70% (Fig. 3b; Additional file 3: Fig. S2A), again with a high degree of consistency in methylation of individual gDMRs determined by the two methods (noting that RRBS and PBAT will not have identical sequence coverage across each gDMR). For example, the *Igf2r* gDMR had attained 32.5% methylation in 50–55 μm oocytes and 67.9% in 60–65 μm oocytes, while the *Cdh15* igDMR was <5% methylated even in 60–65 μm oocytes (Fig. 3b). This range of methylation is broadly consistent with earlier studies that analysed limited numbers of gDMRs by locus-specific bisulphite sequencing (again, with the caveat that different regions of the gDMRs will have been assayed by the various methods; [12–14]). For a subset of CGIs, we also validated the time of onset by locus-specific bisulphite sequencing (Fig. 3c). The differential onset of CGI methylation is not related to CpG content or GC richness of these CGIs (Additional file 3: Fig. S2B). In conclusion, CGIs destined for methylation in GV oocytes are not co-ordinately methylated but display substantial and reproducible differences in time of onset of methylation in growing oocytes, and this variation is not a simple property of overall sequence composition.

### Mapping changes in the oocyte transcriptome during oocyte growth

We sought to test the relationship between methylation kinetics and changes in transcription during oocyte development and growth. To do so, we generated deep, strand-specific total RNA-seq libraries in duplicate from the same size populations of growing oocytes as used in methylation analysis, as well as an earlier population (10–30 μm) and a GV population (Additional file 5: Table S4). In addition, the data were compared with RNA-seq from NGOs collected at embryonic day E18.5 [20] and an existing GV data set [6]. Although transcriptional changes have been documented during mouse oocyte development before [16], those data were generated using expression microarrays that capture only a fraction of the transcription units actually present in oocytes and cannot be used to infer alternative transcription start site (TSS) use: our previous work has demonstrated the importance of using the correct transcriptome for accurate association with methylation [6]. Although the RNA-seq data sets do not capture nascent transcription events, they do enable us to determine the time during oocyte growth that transcription units are first active, including the use of alternative upstream TSSs that are prevalent in oocytes [6]. Transcript abundance was used as a proxy for transcription rate.

**Fig. 4:**
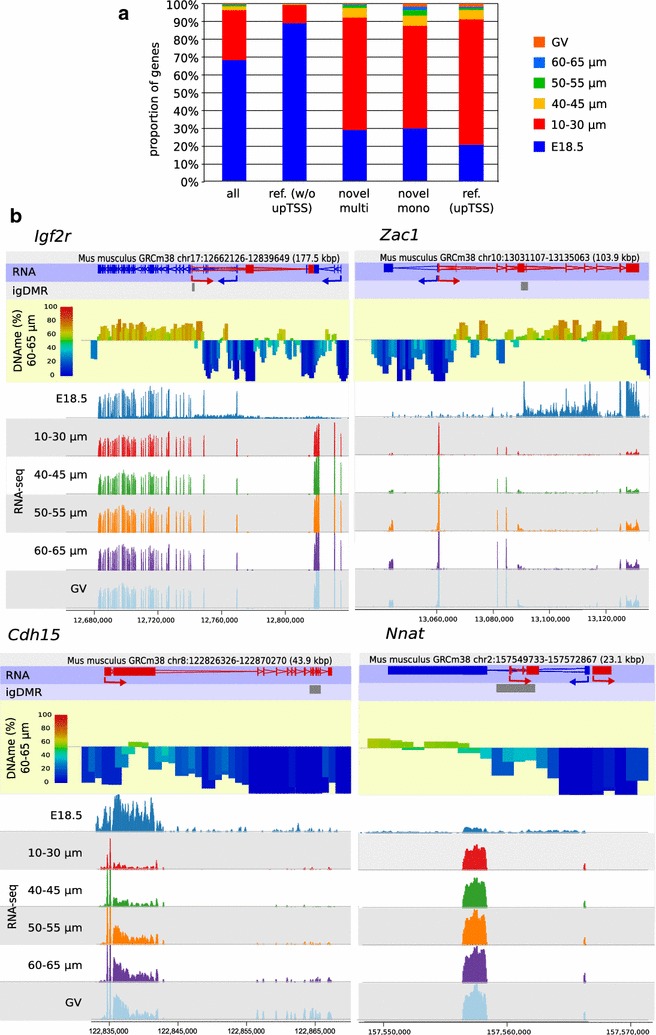
Transcription dynamics in growing oocytes. **a** *Barchart* showing time of first detection of genes in growing oocytes, according to classification as reference gene, from canonical TSS (w/o upTSS) or novel upstream TSS (upTSS), or novel multi- or monoexonic gene. The total numbers of genes classified as expressed in each RNA-seq datasets are given in Additional file 6: Table S5. **b** Browser screenshots of representative early-methylating (*Igf2r*, *Zac1*) and late-methylating (*Cdh15*, *Nnat*) gDMRs in relation to RNA-seq data from different stages of the oocyte growth and DNA methylation acquired in 60–65 µm oocytes. In the RNA annotation track, *red gene* structures are transcribed from *left to right* and *blue gene* structures from *right to left*, with *arrows* showing the most upstream TSSs and direction of transcription. RNA-seq data show that transcriptional pattern is established prior to DNA methylation establishment

The RNA-seq data sets were compared with the oocyte transcriptome assembly previously generated in our laboratory [6], resulting in the detection of 21,402–32,775 genes (FPKM thresholds 0.017–0.102) in the various oocyte size populations (Additional file 6: Table S5). Principal component (PC) analysis of the global expression patterns showed that data sets from growing and GV oocytes cluster together, with the E18.5 transcriptome being the most distinct; PC2 segregates the growing oocyte populations by size, particularly when the E18.5 data set is excluded (Additional file 3: Fig. S3). It should be noted that E18.5 oocytes were collected using FACS, such that RNA was extracted from fixed samples, whereas all post-natal oocytes were collected manually, and these technical differences could contribute to some differences between the E18.5 transcriptome and the other stages. Nevertheless, most transcripts (68%) were already detected at E18.5, and a further 28% were detected first in 10–30 µm oocytes, with very few appearing for the first time in larger size populations (Fig. 4a). The general stability of gene expression in the growing oocyte populations, even as cytoplasmic volume and mRNA content are increasing substantially, is reflected in the rather small numbers of genes identified as differentially expressed (<4%) between consecutive stages (Additional file 3: Fig. S4). Based on our oocyte transcriptome assembly, we segregated genes into reference genes (i.e. previously annotated genes) and novel genes, either novel multiexonic or monoexonic. For reference genes expressed from their canonical TSSs, 88% were already detected at E18.5; in comparison, most novel genes were detected first in 10–30 µm oocytes (~63% multi- and ~57% monoexonic novel genes), with a small minority first detected in larger oocytes (~8 and ~13% for multi- and monoexonic genes, respectively; Fig. 4a). Similarly, most (~70%) novel upstream TSSs were activated in 10–30 µm oocytes. Therefore, most changes in the oocyte transcriptome occur well in advance of the onset of de novo methylation, which initiates after the 40–45 µm stage. This effect can be seen at individual imprinted loci: all gDMRs are found within transcription units even at the earlier stages, irrespective of whether they are transcribed from alternative promoters or whether methylation is detected early (50–55 µm) or late in oocyte growth (60–65 µm) (Fig. 4b).

Despite the general stability of gene expression during oocyte growth stages (Additional file 3: Fig. S4), the RNA-seq data sets provide unprecedented detail into the changes in transcript abundance during critical times in oocyte growth and follicular differentiation. We identified 530 genes, mostly protein-coding, up-regulated greater than 50-fold between E18.5 and GV oocytes, and 283 up-regulated >50-fold between E18.5 and 10–30 μm oocytes (Additional file 7: Table S6). Gene ontology (GO) analysis did not reveal particularly strong enrichment terms (“Regulation of reproductive process” containing 10 of the 283 genes had the highest enrichment of 5.53, *p* value 1.27 × 10^−5^, adjusted FDR 0.164), perhaps reflecting the wide diversity of functions required during oogenesis as well as the accumulation of maternal RNA stores for processes in the zygote (Additional file 3: Fig. S5). The set of highly induced transcripts did contain genes for oocyte-specific transcriptional regulators such as OBOX1, 2 and 5, the maternal effect homeobox SEBOX, the zona pellucida proteins 1, 2 and 3 (ZP1, 2, 3), components of the sub-cortical maternal complex (OOEP, TLE6) and members of the reproduction-related NLRP family (nucleotide-binding oligomerization domain, leucine-rich repeat and pyrin domain-containing proteins), as well as oocyte genes with less well explored functions (*Oas1d*, *Oosp1*, *Omt2b*) (Additional file 7: Table S6). We also specifically examined the gene expression dynamics of candidate factors involved in de novo DNA methylation and associated epigenetic modifications, such as DNMT3A and DNMT3L, H3K4 demethylases of the KDM1 and KDM5 families, and the H3K36 methyltransferase SETD2. Although many of the corresponding genes appear to be stably expressed during oocyte growth, there was substantial up-regulation of *Kdm1b*, *Dnmt1* and particularly *Dnmt3L*, whose transcripts appear first in 10–30 μm oocytes (Additional file 3: Fig. S6). These transcript dynamics are consistent with the reported appearance of KDM1B and DNMT3L proteins during oocyte growth [21, 30].

### DNA methylation kinetics in relation to transcription events

**Fig. 5:**
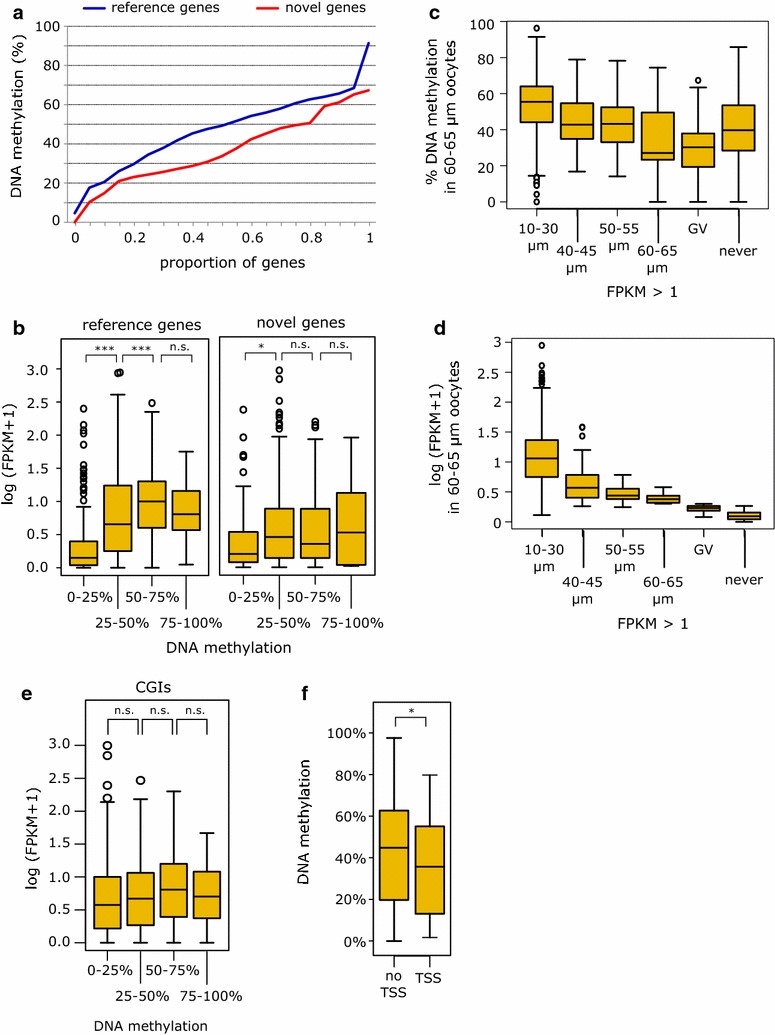
Gene body and CpG island methylation kinetics in relation to transcription. **a** Cumulative distribution plot of methylation level of reference and novel genes (≥4 kb in length and ≥2 FPKM) in 60–65 µm oocytes (PBAT dataset). The numbers of reference and novel genes satisfying the criteria for analysis were 105 and 32, respectively. **b** *Box whisker plots* of methylation of gene bodies of reference (1396) and novel (373) genes in relation to expression level in 60–65 µm oocytes. **c** *Box whisker plot* showing methylation level of CGIs in 60–65 µm oocytes grouped according to the stage in oocyte growth that expression of overlapping gene attained the threshold of >1 FPKM in the RNA-seq datasets. **d** *Box whisker plot* showing the corresponding data from expression level in 60–65 µm oocytes. The numbers of genes in (**c**) and (**d**) are: 1013 for 10–30 µm oocytes, 76 for 40–45 µm, 70 for 50–55 µm, 47 for 60–65 µm, 57 for GV and never 289. **e** Methylation level of intragenic CGIs (CGIs fully methylated in GV oocytes) in relation to expression level of the corresponding gene in 60–65 µm oocytes. The numbers of CGIs analysed in each methylation category (from lowest to highest) are: 269, 210, 281 and 50. **f** Methylation in 60–65 µm oocytes of CGIs (CGIs fully methylated in GV oocytes) according to prior activity as TSS as determined in e18.5 oocytes: 112 TSS-CGIs and 1229 non-TSS-CGIs. *Asterisks* denote *p* values of Student’s t test: *0.01–0.001, **0.001–0.0001, ***<0.0001

Although the global results above do not support a major role for activation of specific transcription units in the timing of de novo methylation, we performed several additional analyses to investigate in more detail possible relationships between transcription events and temporal control of methylation. We compared the methylation level of multiexonic reference genes and multiexonic novel genes, reasoning that the reference genes are generally expressed from earlier time points in oocyte growth (Fig. 4a). For this, we selected genes ≥4 kb in length (as shorter genes are unmethylated across much of their length) and set an expression threshold of ≥2 FPKM (to mitigate an effect of expression level). In this comparison, reference genes as a set have accumulated more methylation in 60–65 µm oocytes (Fig. 5a). Level of expression could still contribute to this effect, as novel genes are less highly expressed [6]: for the genes we included above 2 FPKM, median FPKM values were 11.4 and 3.9 for reference and novel genes, respectively. Indeed, there was a positive correlation between gene body methylation and expression level in 60–65 µm oocytes, particularly for reference genes, although the relationship plateaus for more highly methylated gene bodies (Fig. 5b). We also considered whether genes exceeding an expression threshold earlier during oocyte growth acquire methylation sooner, and this appeared to be the case (Fig. 5c). Again, however, it is difficult to separate out an effect of gene expression level, as genes crossing the threshold earlier are also more highly expressed in 60–65 µm oocytes (Fig. 5d). An effect on host gene expression was apparent for intragenic CGIs that gain methylation during oocyte growth, although the differences between groups were not significant (Fig. 5e). We also examined whether the extent of methylation of these CGIs in 60–65 µm oocytes reflected whether they were active TSSs at an earlier stage (E18.5 NGOs). Indeed, CGIs previously acting as TSSs had gained less methylation on average than non-TSS-CGIs (Fig. 5f). This analysis was performed with the PBAT data set, as RRBS data have limited coverage of gene bodies. When we compared DNA methylation of intragenic CGIs in 50–55 and 60–65 µm RRBS data sets with expression levels of overlapping genes in the corresponding RNA-seq datasets, we obtained similar results to the PBAT data (Additional file 3: Fig. S7).

**Fig. 6:**
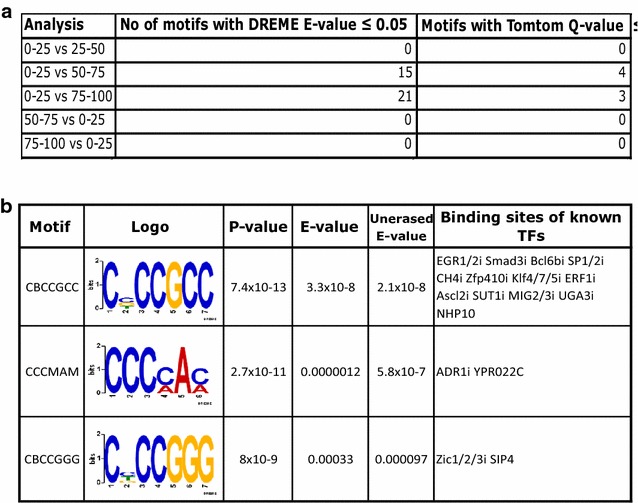
Motifs differentially enriched in early- and late-methylating CpG islands. **a** Summary of results of DREME analysis identifying motifs differentially enriched in CGIs that become fully methylated in GV oocytes grouped according to methylation level in 60–65 µm oocytes. Codes 0–25, 25–50, 50–75 and 75–100 represent CGIs with corresponding percentage methylation in 60–65 µm oocytes. The numbers of CGIs in each category are 470, 329, 384 and 63, respectively. **b** DREME motifs significantly enriched in CGIs methylated ≤25% in oocytes compared with ≥75% methylated CGIs that correspond to binding site motifs for known TFs. In motif sequence, *B* = C/G/T and *M* = C/A. *P* value and *E* values are as defined by DREME and binding sites as identified by Tomtom

Changes in TSS use could reflect changes in binding of sequence-specific TFs at these sites, possibly as a consequence of down- or up-regulation of these factors during oocyte growth. In this context, it has previously been reported that the CGCGC consensus site of E2F1 and E2F2 is enriched in intragenic CGIs that are completely resistant to de novo methylation in oocytes [31]. Accordingly, we used the motif analysis package DREME [32] to identify motifs differentially enriched in CGIs with different levels of methylation in 60–65 µm oocytes. We searched for motifs enriched in late-methylated CGIs (≤25%) compared to CGIs with 25–50, 50–75 and ≥75% methylation, as well as for motifs enriched in early-methylated CGIs (≥75 and 50–75% methylation) compared to late-methylated CGIs (Fig. 6a). There were no motifs enriched in early-methylated CGIs compared to CGIs gaining methylation later, suggesting that there is no sequence motif targeting methylation to specific CGIs. On the other hand, we found motifs significantly enriched in late-methylated CGIs. Considering the comparison between ≥75% methylated and ≤25% methylated CGIs as likely to give the greatest discriminating signal, there were 21 sequence motifs with a significant difference in enrichment, three of which correspond to binding sites of known TFs (Fig. 6b; Additional file 8: Table S7). Of these, the most significant motif C(C/G/T)CCGCC (p value = 7.4 × 10^−13^) was detected in 55% of the late-methylating CGIs but only 9.5% of the early-methylating CGIs. We repeated the analysis with the MEME motif analysis package [33] to search for longer motifs than DREME. Again, the significantly enriched motifs were found only in late-methylated CGIs compared to CGIs with methylation of 50–75 and ≥75%. Late-methylated CGIs appear to be enriched in G-rich motifs; however, these motifs are also present in 50% or more of the early-methylating CGIs (Additional file 3: Fig. S8).

### CGI methylation in relation to chromatin state

**Fig. 7:**
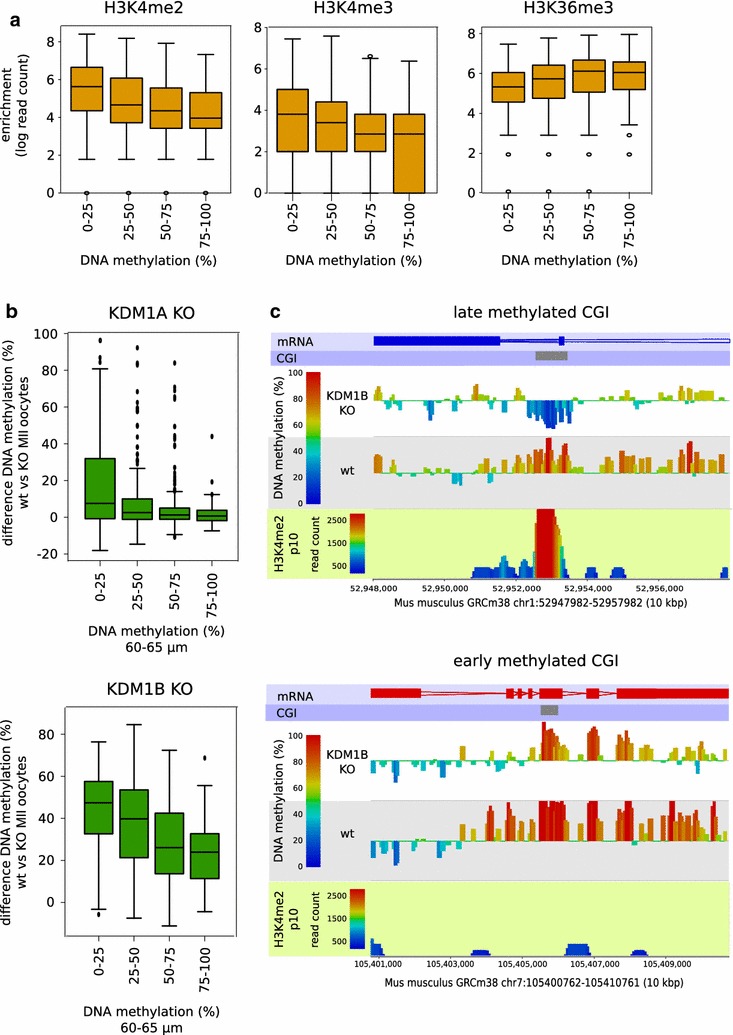
CpG island methylation kinetics in relation to chromatin parameters. **a** *Box whisker plots* showing enrichment (log-transformed corrected read count) of H3K4me2, H3K4me3 and H3K36me3 at CGIs in relation to DNA methylation in 60–65 µm oocytes (PBAT data). The ChIP-seq data shown are from p10 oocytes; similar trends were observed in ChIP-seq data from e18.5 oocytes. Pearson’s correlation coefficients are: −0.293 for H3K4me2, −0.173 for H3K4me3, 0.240 for H3K36me3. The numbers of CGIs analysed in each methylation category (from lowest to highest) were: 464, 327, 382 and 63. **b** *Box whisker plots* showing the degree of DNA methylation change at CGIs in *Kdm1a*- and *Kdm1b*-null MII oocytes in relation to methylation in 60–65 µm oocytes. Pearson’s correlation coefficients are: −0.296 for *Kdm1a* and −0.357 for *Kdm1b*. The numbers of CGIs analysed in each methylation category (from lowest to highest) were: 244, 185, 255 and 28 for *Kdm1a*, and 270, 199, 268 and 31 for *Kdm1b*. **c** Browser screenshots of a representative early-methylating and late-methylating CGI (84.2 and 12.2% methylation in 60–65 µm oocytes, respectively) in relation to p10 H3K4me2 enrichment and DNA methylation attained in wild-type (WT) or *Kdm1b*-null MII oocytes

Since transcription does not appear to be an overriding factor in the differential timing of CGI methylation, we examined the influence of specific histone post-translational modifications, given the likely importance of chromatin state in recruitment of the DNMT3A:DNMT3L complex. We divided CGIs that become fully methylated (≥75%) in GV oocytes into levels of methylation attained in 60–65 μm oocytes and assessed the enrichment of histone modifications as determined by chromatin immunoprecipitation and sequencing (ChIP-seq) in NGOs (isolated at E18.5) and early growing oocytes (post-natal day p10) [20]. Of the modifications implicated in promoting or antagonising DNA methylation, levels of H3K36me3 showed a positive correlation with DNA methylation level; H3K4me2 and H3K4me3, conversely, were negatively correlated (Fig. 7a, all p values <1 × 10^−10^). We then looked whether there was a relationship with dependence on the H3K4me2 demethylases KDM1A and KDM1B. We have previously shown that loss of KDM1B, in particular, affects the methylation level acquired in MII oocytes of many CGIs, but there is a considerable variation in the magnitude of the dependency [20]. Therefore, we compared the change in DNA methylation of CGIs in oocytes deficient in KDM1A or KDM1B with level of methylation in wild-type, 60–65 μm oocytes, which showed that later-methylating CGIs (i.e. less methylation in 60–65 μm oocytes) are most dependent on KDM1A or KDM1B to become fully methylated in MII oocytes (Fig. 7b). Examples of early- and late-methylating CGIs in relation to H3K4me2 level and KDM1B dependence are shown in Fig. 7c.

### Modelling factors determining rate of CGI methylation

To test the extent to which the above variables, alone or in combination, account for the differential timing of CGI methylation in growing oocytes, we applied several regression models. We considered up to nine independent variables, including the three transcription factor binding motifs significantly enriched in the late-methylating CGIs (Table 1), with methylation level in 60–65 µm oocytes as response variable. As all the variables except GC content are in statistically significant linear relationship with the response variable, we first tested how much of the methylation variation could be attributed to each of the variables alone in simple linear regression models. H3K4me2 enrichment at p10 and dependence on KDM1B and KDM1A explained the highest proportion of the variability in the methylation data: 11.2, 10.5 and 9.7%, respectively.

**Table 1:** Linear regression models explaining DNA methylation level at CGIs in 60–65 µm oocytes

Variable	Simple linear regression		Lasso regression
	Significance (*p* value)	% variability explained	Coefficient value in the most regularised model^b, c^
H3K4me3 enrichment, p10 ChIP-seq	2.34 × 10^−6^	4.8	N/A
H3K4me2 enrichment, p10 ChIP-seq	2.88 × 10^−13^	11.2	−0.132
H3K36me3 enrichment, p10 ChIP-seq	8.21 × 10^−8^	6.2	0.050
KDM1A dependence	1.1 × 10^−11^	9.7	−0.132
KDM1B dependence	1.5 × 10^−12^	10.5	−0.106
CpG density	0.000767	2.5	N/A
%GC content	0.169199	0.9	N/A
Transcription level (log-transformed)	0.000297	2.9	N/A
Enriched motif occurences (CBCCGCC, CCCMAM, CBCCGGG^a^)	3.97 × 10^−8^	6.5	−0.019

Simple linear regressions (variables tested individually) and multiple linear regression (variables tested together) modelling the relationship between explanatory variables and DNA methylation level at CGIs in 60–65 µm oocytes. The outcome of the model is presented as a proportion of the variability in DNA methylation level at CGIs in 60–65 µm oocytes explained by the variables

^a^See Fig. 6a for motifs details. These three motifs were selected as they represent binding sites of known proteins

**Fig. 8:**
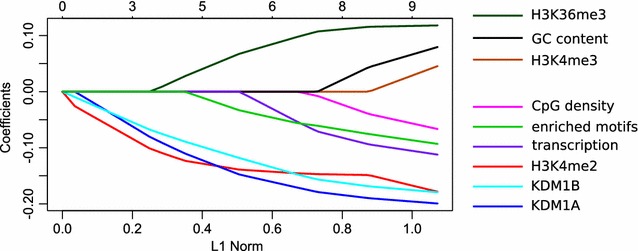
Modelling factors determining rate of CpG island methylation. Lasso regression model plot showing the effect of nine independent variables on variability of CGI methylation in 60–65 µm oocytes. *Each line* represents one of the variables. The earlier the line deviates from the *horizontal line* with coefficient 0.0, the more the corresponding variable contributes to the variability of the response variable, and the steeper the slope the greater the effect. If the steepness of the slope of one variable already in the model changes when a new variable comes into the model, it is a sign of correlation between two independent variables

^b^Coefficients of variables in the model selected after software cross-validation of models as the most regularised model. These coefficients correspond to the values on y axis in Fig. 8. N/A marks variables that are not included in the model

^c^The Lasso regression model including the 5 variables indicated in the column accounts for 18.5% of the variation

Because of the multicollinearity amongst independent variables (e.g. high correlation between transcription level and H3K36me3 enrichment, or between H3K4me2 and H3K4me3 enrichments), we could not test the combination of all variables in a classical multiple linear regression model. Instead, we applied linear modelling approaches correcting for multicollinearity—Ridge, Lasso and ElasticNet regressions—and looked for the best fit. Lasso and ElasticNet regression models using all nine variables explain 23.14% of the variability (Fig. 8). However, the cross-validation of models, where individual independent variables are added one by one to the model, in each step adding the variable that explains the highest proportion of the variability, revealed that H3K4me2 enrichment, KDM1A and KDM1B dependency, H3K36me3 enrichment and the presence of TF binding motifs are sufficient for the model, explaining 18.5% of the variability (Table 1). Although the remaining variables increase the explained proportion of methylation variability, they also increase the noise level and therefore do not statistically improve the model. We also tested other regression modelling approaches not requiring the linear relationships, such as polynomial regression; however, the fit of the models was not improved.

## Discussion

DNA methylation in the mouse oocyte depends upon DNMT3A and DNMT3L is primarily over gene bodies and largely determined by transcription, but these global dependencies could obscure sequence-specific requirements or the involvement of additional factors at specific elements. By capturing oocytes in the mid-phase of de novo methylation, we find that all sequence features gain CpG methylation at similar rates overall, including most classes of TEs, suggesting a universal rather than a feature-specific targeting mechanism. CGIs as a whole and a subset of L1 elements gain methylation later, however. In relation to CGIs, this relative delay might reflect that they are marked by default with histone modifications antagonistic to DNA methylation, such as H3K4me2/me3, and younger L1 elements may be suppressed by histone modifications inhibitory to DNA methylation. Amongst CGIs, however, there are reproducible differences in time of onset and/or progression in de novo methylation. This finding, at the genome-wide scale, substantially extends earlier studies on limited numbers of imprinted gDMRs [12–14] and suggests that CGIs destined for methylation initially exist in different states of permissiveness. There are a number of factors that could contribute to this asynchrony. Nuclear availability of DNMT3A [34] and DNMT3L is an absolute requirement, and DNMT3L is potently up-regulated during oocyte growth. A study in which DNMT3A2 and DNMT3L were precociously expressed in oocytes was not able to induce methylation of imprinted gDMRs in NGOs however, but did advance methylation of some gDMRs in growing oocytes [30], indicating that some loci are in a state permissive for methylation earlier than others.

Having established a major role for transcription in conferring the DNA methylation landscape of the oocyte, including at CGIs [5–7], we reasoned that timing of transcription events could influence timing of methylation. In fact, we did not find strong evidence to support this proposition. Despite substantial transcriptional changes during initial stages of oocyte growth, most changes occur well in advance of the onset of methylation, indicating that remodelling of the oocyte transcriptome is not a rate-limiting step in determining permissiveness of individual loci. We did find a positive correlation between expression and methylation however, so more highly expressed gene bodies on average gain methylation earlier than less highly expressed genes; this effect could be mediated through transcription-depending chromatin remodelling, including deposition of H3K36me3, whose levels over gene bodies scale with expression in oocytes [20]. A caveat to our analysis is that we used transcript abundance as measured by RNA-seq as a proxy for transcription, rather than directly determining active transcription events. This is because methods have not been developed to allow nascent transcription (such as by NET-seq) to be mapped in small numbers of cells. However, at the very least, the RNA-seq data allow us to determine the time that genes are first transcribed during oocyte growth.

To explain the difference in onset of methylation at CGIs, we considered the contributions of up to nine variables for which data were available. In combination, these variables explain about a fifth of the variation in timing of methylation establishment, with chromatin factors—H3K4me2 enrichment, KDM1A dependence and KDM1B dependence—having the greatest individual effects. There may be several reasons that we are not able to account for more of the variation at this time, apart from unknown factors not included in the modelling. One reason might be the relative imprecision in some of the data types; for example, low-cell ChIP-seq data for histone modifications in growing oocytes are inherently noisy, being at the limits of the capability of this method, and will have missing values at some CGIs. In comparison, PBAT data from *Kdm1a*- and *Kdm1b*-null MII oocytes are likely to be more precise. Therefore, it is reassuring that the magnitude of the individual effects of H3K4me2 enrichment and KDM1B dependence is so similar, since these are likely to be partially dependent variables given that we previously concluded that KDM1B is the major locus-specific H3K4me2 demethylase in oocytes [20]. It was previously suggested that KDM1B may be required to allow methylation of imprinted gDMRs that acquire methylation late in oocyte growth [21]; our genome-wide analysis and modelling partly support this earlier inference.

Gross sequence composition accounts for little of the variation in CGI methylation timing. Although CpG density is a determinant of H3K4me2 enrichment at CGIs, CGIs destined for methylation in oocytes are relatively depleted of H3K4me2 irrespective of CpG density [20]. Several sequence motifs, however, were differentially represented in early- and late-methylating CGIs. Individually, these motifs are not as discriminating as the ZFP57 binding site in imprinted gDMRs [35] that ensures retention of methylation after fertilisation, or the E2F1/E2F2 motif enriched in CGIs that escape DNA methylation in oocytes [31]. When combined, the three motifs for known TFs explain about half as much of the variation in methylation onset as do each of the chromatin factors. These motifs correspond to binding sites for 15 TFs expressed at varying levels in oocytes. Although some of their transcripts are down-regulated during oocyte growth (Additional file 9: Table S8), it is not possible at this stage to conclude whether the dynamics of any of these TFs underlies the differential methylation onset of the CGIs.

By capturing the progression of methylation, we also reveal other important aspects of de novo methylation in an in vivo setting, extending the significance of studies done in models such as ESCs. For example, we identify a co-operativity and nucleosomal pattern of DNMT3A action similar to that observed in ESCs [28]. Non-CpG methylation has been described as a property of oocytes as well as other non-dividing cells [11, 29, 36], but remains an enigmatic modification. Even in oocytes, in which methylation globally at CHG and CHH sites exceeds that at CG sites, few non-CpG sites are methylated (genome-wide average methylation of CHGs is 3.9%, and CHHs are 3.0% compared with 38.7% at CG sites as quantified with our parameters using published data [11]), with sites methylated mostly only to intermediate levels; moreover, CHH/CHG methylation is highly associated with domains of CpG methylation. Combined with its very much later onset, this suggests that CHG/CHH methylation is largely a by-product of sustained DNMT3A activity. Finally, DNA methylated domains not associated with transcribed regions are also late in acquiring methylation, suggesting that they require additional remodelling steps or a distinct mechanism of de novo methylation.

## Conclusions

The mammalian oocyte provides an important model to understand DNA methylation mechanisms, because an entire methylation landscape is established de novo in a non-dividing cell. Epigenetic remodelling events culminate in a distinctive DNA methylation landscape, including the programmed methylation of a defined set of CGIs, mostly associated with transcription units. Despite the simplicity of the methylation landscape, various sequence elements are not co-ordinately methylated, with pronounced asynchrony in methylation of CGIs. In this study, we generated methylation and transcriptome data sets to test whether timing of transcription events explained asynchrony of CGI methylation; however, our results do not support transcriptional transitions as a major factor in time of onset of methylation. By incorporating data on chromatin state, TF binding motifs and the effect of deficiency in H3K4 demethylases, we could account for a substantial fraction of variation in CGI methylation timing, suggesting that sequences least dependent on chromatin remodelling are the earliest to become permissive for methylation.

## Methods

### Isolation and size selection of growing oocytes

Oocytes were collected from C57BL/6Babr mice. Ovaries were removed and digested for 30 min at 37 °C in 1× PBS containing 2 mg/ml collagenase (Sigma-Aldrich, C2674) and 0.025% trypsin (Sigma-Aldrich, 93615). M2 medium (Sigma-Aldrich, M7167) was added to dilute the digestion mix, and oocytes were picked up with a mouth-controlled drawn-out glass pipette. To eliminate contaminating somatic cells, oocytes were washed extensively in clean drops of M2 medium. A stage micrometre was used in combination with an eyepiece reticle to measure sizes of oocytes. Mice of post-natal days p5–7, p7–12, p9–14 and p13–16 were used to collect oocytes of 10–30, 40–45, 50–55 and 60–65 µm in diameter, respectively; GV oocytes were collected at p20.

### Generation of PBAT and RRBS libraries

RRBS libraries were generated, in duplicate, from ~450 to 550 oocytes per size-selected population, as previously described [7], but without the gel-extraction step. Briefly, DNA was spiked with a small amount of lambda DNA (0.05 pg per 6 ng genomic DNA) for bisulphite conversion control, digested with MspI (Thermo Fisher Scientific, ER0541), end-repaired (Klenow fragment exo-, Thermo Fisher Scientific, EP0421, with 10 nM dATP, 1 nM dCTP and 1 nM dGTP) and ligated with 5mC-adapters (Illumina) with T4 ligase (Thermo Fisher Scientific, EL0014). Bisulphite conversion was done using the EZ DNA Methylation-Direct Kit (Zymo Research, D5020), and DNA was amplified by 18 cycles of PCR using PfuTurbo Cx Hotstart DNA polymerase (Agilent, 600410). Libraries were purified using SPRI beads (Agencourt, A63880) and sequenced 40 bp single end on an Illumina Genome Analyzer IIx. The PBAT library was constructed from 200 60–65 µm oocytes as previously described [20] and sequenced 100 bp paired end on an Illumina HiSeq 1000.

### Generation of strand-specific RNA-seq libraries

Strand-specific RNA-seq libraries were generated as previously described [6] and sequenced 100 bp paired end on an Illumina HiSeq 1000. The numbers of oocytes used per library are listed in Additional file 5: Table S4.

### Conventional bisulphite sequencing

Bisulphite sequencing was performed essentially as previously described [29] using DNA from ~100 to 200 oocytes plus 50 ng lambda DNA spike-in for each bisulphite conversion using the EZ DNA Methylation Kit (Zymo Research, D5001). Bisulphite-converted DNA from >30 oocytes was used for each PCR amplification; primers are listed in Additional file 10: Table S9. PCR products were cloned using pGEM-T Easy Vector Systems (Promega, A1360) and sequenced with the universal M13 primer. Experiments were done in duplicate for each size group and results combined.

### Mapping sequence reads

RRBS reads were trimmed to remove poor quality calls and adapters using Trim Galore v0.3.5 (parameters –rrbs) and mapped to the mouse genome GRCm38 assembly by Bismark [37] v0.14.0 (options –phred64-quals). For PBAT data, trimmed reads (Trim Galore v0.3.5 using default parameters) were first aligned to GRCm38 in paired-end mode to count overlapping parts of the reads only once while writing out unmapped singleton reads; in a second step remaining singleton reads were aligned in single-end mode. Alignments were carried out with Bismark v0.10.0 with the following parameters: –pbat for paired-end mode, –pbat for single-end mode for read 1 and default parameters for single-end mode read 2. Reads were then deduplicated with Bismark selecting a random alignment for positions covered more than once. CG, CHH and CHG methylation calls were extracted using the Bismark methylation extractor (v0.10.0) with the parameters: –no_overlap –report –ignore 4 –ignore_r2 4 for paired-end mode and –report –ignore 4 for the single-end mode. Published bisulphite-sequencing data were processed as described previously [6]. Raw RNA-seq reads were trimmed to remove poor quality calls and adapters using TrimGalore v.0.2.8 and mapped to GRCm38 using TopHat v.2.0.9 (option –g 1).

### Data analysis and modelling

We used reference and oocyte transcriptomes defined previously [6], including the definition of novel genes and novel TSSs of known genes, coordinates of CGIs, imprinted gDMRs, TEs and methylation domains. CGIs and TEs were used for methylation analyses if the minimum number of reads to count a position/minimum number of positions to count a probe were 5/5 for CGIs, 5/3 for all TEs and 3/3 if only methylated or unmethylated TEs were analysed. Otherwise, informative Cs refers to one covered by a minimum of 5 reads. Concordance of methylation of adjacent CpGs was quantified using custom Perl scripts, using CpGs with ≥5 reads. Expression of transcripts was quantified using Cufflinks v2.1.1 with –G option. Expression of genes was determined as a sum of FPKM values of all transcripts per gene. Expression of upstream TSSs and FPKM cut-off values to discriminate expressed and silent transcripts was defined as previously [6]. A gene was classified as expressed if at least one of its isoforms was classified as expressed. A gene/TSS was classified as activated at a specific stage if it was classified as expressed in both replicates of that stage, as silent in both replicates in previous stages and as expressed in both replicates in subsequent stages. DNA methylation, RNA-seq and ChIP-seq data were analysed using SeqMonk v0.29.0–0.34.0. PC analysis and statistical analyses were performed in R v.3.0.2. Motif enrichment analysis was performed using DREME [32] and MEME [33] within the MEME suite v4.11.1 with default parameters specifying list of control sequences. Enriched motifs were directly submitted to Tomtom [38] within the MEME suite using default parameters. Regression modelling the relationship between CGI methylation in 60–65 µm oocytes and the variables listed in Table 1 was performed in R v.3.0.2 using CGIs with all information available. Function lm was used for linear regression, package glmnet for Lasso, Ridge and ElasiticNet regression, including cross-validation of models. Values of response and independent variables were normalised to mean 0 and standard deviation 1. GO analysis was performed using GOrilla [39] with specifying all genes expressed in the oocytes as a background.

## Additional files

**Additional file 1: Table S1.** Sequencing statistics for PBAT and RRBS libraries from size-selected oocytes.

**Additional file 2: Table S2.** Methylation levels of all CpGs and various genomic features that become methylated in GV oocytes in NGO, 60–65 µm and GV oocytes.

**Additional file 3: Figure S1.** Progression of DNA methylation at transposable elements (TEs) during oocyte growth, complements Fig. 1. **Figure S2:** Methylation parameters in 60–65 µm oocytes, including comparison of PBAT and RRBS data sets. **Figure S3.** PCA plots for oocyte mRNA-seq libraries. **Figure S4.** Differentially expressed genes between consecutive oocyte size populations. **Figure S5.** GO analysis of genes up-regulated >50-fold between e18.5 and GV oocytes. **Figure S6.** Graph showing expression levels over oocyte growth of transcripts for Dnmts, Kdm1s and Kdm5s, and SetD2. **Figure S7.** Methylation level of intragenic CGIs in RRBS datasets in relation to expression level of the corresponding gene. **Figure S8.** MEME output of search for motifs enriched in late-methylated CGIs.

**Additional file 4: Table S3.** CG, CHG and CHH methylation levels in NGO, 60–65 µm and GV oocytes.

**Additional file 5: Table S4.** Sequencing statistics for ssRNA-seq libraries from NGO, size-selected and GV oocytes.

**Additional file 6: Table S5.** Numbers of genes detected in ssRNA-seq libraries.

**Additional file 7: Table S6.** Genes up-regulated ≥50 from e18.5 to 10–30 µm oocytes and from E18.5 to GV oocytes.

**Additional file 8: Table S7.** Output from DREME analysis of sequence motifs differentially represented in early- and late-methylating CpG islands.

**Additional file 9: Table S8.** Expression levels of transcription factors with binding motifs enriched in CGIs with low methylation level in 60–65 µm oocytes.

**Additional file 10: Table S9.** PCR primers sequences for conventional bisulphite sequencing of selected CGIs.

## Abbreviations

*Cdh15*: cadherin 15 gene
CGIs: CpG islands
ChIP-seq: chromatin immunoprecipitation with high-throughput sequencing
DNMT3A/B/L: DNA methyltransferase 3A, 3B or 3L
DREME: discriminative regular expression motif elicitation
E18.5: embryonic day 18.5
E2F1/2: E2F transcription factor 1 or 2
ERVK: endogenous retrovirus, K family
ESC: embryonic stem cell
FDR: false discovery rate
FPKM: fragments per kilobase per million mapped reads
gDMR: germline differentially methylated region
GO: gene ontology
GV: germinal vesicle
H3K4: histone 3 lysine residue 4
H3K4me2/me3: di- or trimethylated H3K4
H3K36me3: trimethylated H3K36
*Igf2r*: insulin-like growth factor 2 receptor
KDM1A/1B: lysine demethylase 1A or 1B
KDM5: lysine demethylase 5 family
LINE: long interspersed nuclear element
LTR: long-terminal repeat
MII: metaphase II
MEME: multiple expectation maximisation (EM) for motif elicitation
NET-seq: native elongating transcript sequencing
NGO: non-growing oocytes
*Oas1d*: 2′,5′-oligoadenylate synthetase 1-like D, gene
OBOX1/2/5: oocyte-specific homeobox 1, 2 or 5
*Omt2b*: oocyte maturation, beta, gene
OOEP: oocyte-expressed protein
*Oosp1*: oocyte-specific protein 1, gene
P10: post-natal day 10
PC: principal component
PBAT: post-bisulphite adapter tagging
RRBS: reduced representation bisulphite sequencing
RNA-seq: RNA sequencing
SEBOX: skin–embryo–brain–oocyte-specific homeobox
SETD2: SET domain-containing 2
SINE: short interspersed nuclear element
TE: transposable element
TF: transcription factor
TLE6: transducing-like enhancer of split
TSS: transcription start site
*Zac1*: zinc finger protein regulating apoptosis and cell cycle arrest, gene
ZFP57: zinc finger protein 57
ZP1, 2, 3: zona pellucida glycoprotein 1, 2 or 3
