# Supplementary material for: Transcription and chromatin determinants of de novo DNA methylation timing in oocytes
Source: Epigenetics Chromatin. 2017 May 12;10:25. doi: 10.1186/s13072-017-0133-5 (PMC5429541; doi:10.1186/s13072-017-0133-5)
Supplement: Supplementary file 3 — Additional file 3: Figure S1. Progression of DNA methylation at transposable elements (TEs) during oocyte growth, complements Fig. 1. Figure S2: Methylation parameters in 60–65 µm oocytes, including comparison of PBAT and RRBS data sets. Figure S3. PCA plots for oocyte mRNA-seq libraries. Figure S4. Differentially expressed genes between consecutive oocyte size populations. Figure S5. GO analysis of genes up-regulated >50-fold between e18.5 and GV oocytes. Figure S6. Graph showing expression levels over oocyte growth of transcripts for Dnmts, Kdm1s and Kdm5s, and SetD2. Figure S7. Methylation level of intragenic CGIs in RRBS datasets in relation to expression level of the corresponding gene. Figure S8. MEME output of search for motifs enriched in late-methylated CGIs. [file 13072_2017_133_MOESM3_ESM.pdf]

Additional file 3: Figure S1

Progression of DNA methylation at transposable elements (TEs) during oocyte growth, complements Figure 1.

(A) Violin plots showing the distribution of CpG methylation values in LINE L2s, LTR-ERV1s, LTR-ERVKs, LTR-ERVLs, LTR-MaLRs and SINE-B4s in NGO, 60-65 µm and GV oocytes. Shape of the violin plot represents Kernel density estimation, i.e., the probability density of the data at the different values. White dots correspond to the median, yellow dots to the average, bold lines represent the interquartile range and thin lines adjacent values, i.e., the minimum and maximum values within the 1.5x interquartile range from the first and third quartile, respectively. (B) Heatmaps of methylation levels of TEs of individual LINE-L1 and LTR-MaLR subfamilies in 60-65 µm oocytes that become methylated (>75%) in GV oocytes. Individual TEs in each subfamily are ordered from lowest to highest methylation level. Between 181 and 4804 elements were analysed in each LINE-L1 subfamily, and between 112 and 1539 elements in each LTR-MaLR subfamily, except MTA_Mm subfamily with only 16 elements fulfilling the criteria. Asterisks denote *p*-values from ANOVA (* 0.01-0.001, ** 0.001-0.0001, ***<0.0001).

Figure S1

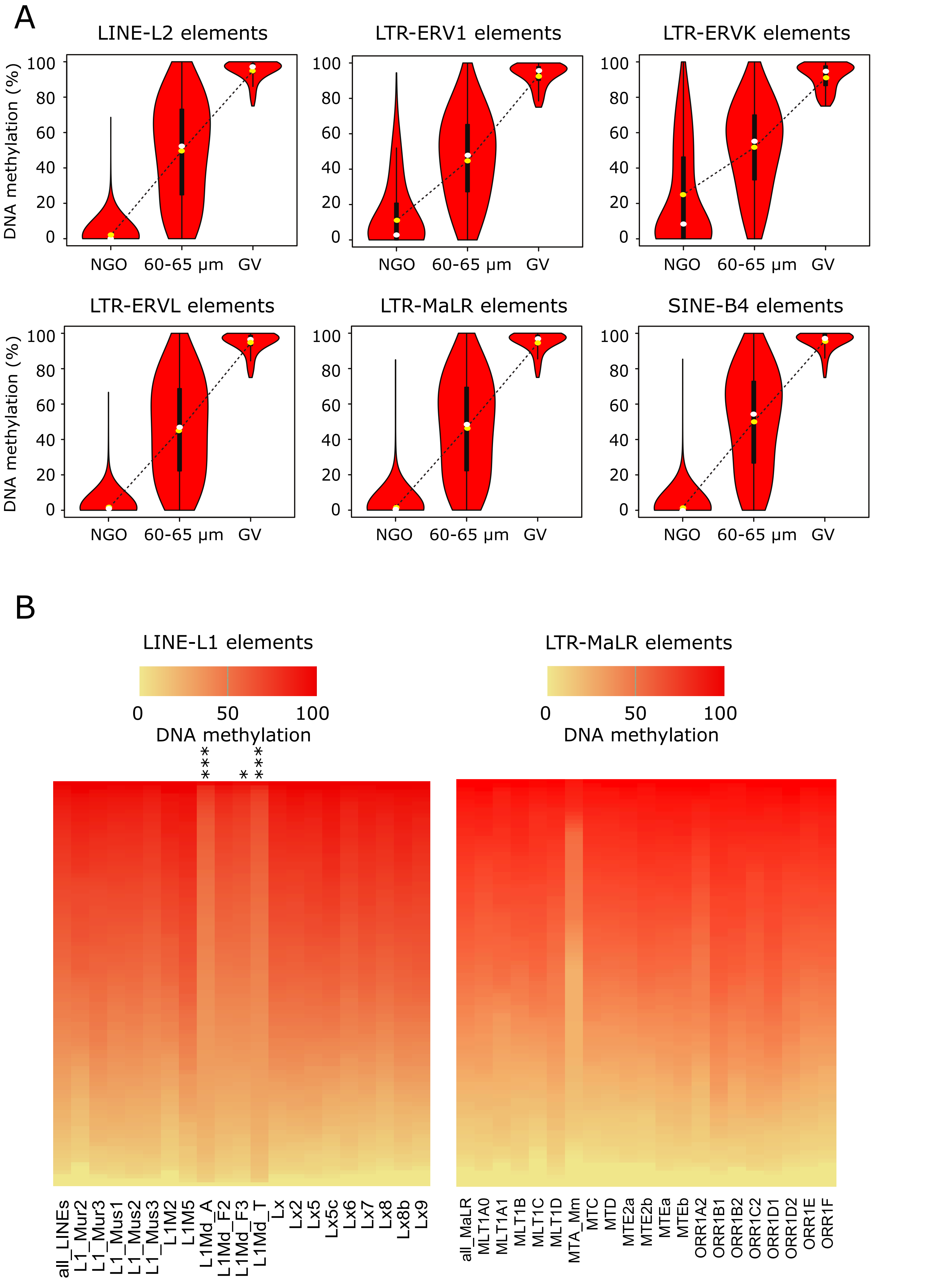
A
LINE-L2 elements
LTR-ERV1 elements
LTR-ERVK elements
LTR-ERVL elements
LTR-MaLR elements
SINE-B4 elements
DNA methylation (%)
0
20
40
60
80
100
NGO
60-65 µm
GV
B
LINE-L1 elements
LTR-MaLR elements
0
50
100
DNA methylation
***
*
***
all_LINEs
L1_Mur2
L1_Mur3
L1_Mus1
L1_Mus2
L1_Mus3
L1M2
L1M5
L1Md_A
L1Md_F2
L1Md_F3
L1Md_T
Lx
Lx2
Lx5
Lx5c
Lx6
Lx7
Lx8
Lx8b
Lx9
all_MaLR
MLT1A0
MLT1A1
MLT1B
MLT1C
MLT1D
MTA_Mm
MTC
MTD
MTE2a
MTE2b
MTEa
MTEb
ORR1A2
ORR1B1
ORR1B2
ORR1C2
ORR1D1
ORR1D2
ORR1E
ORR1F

Figure S2

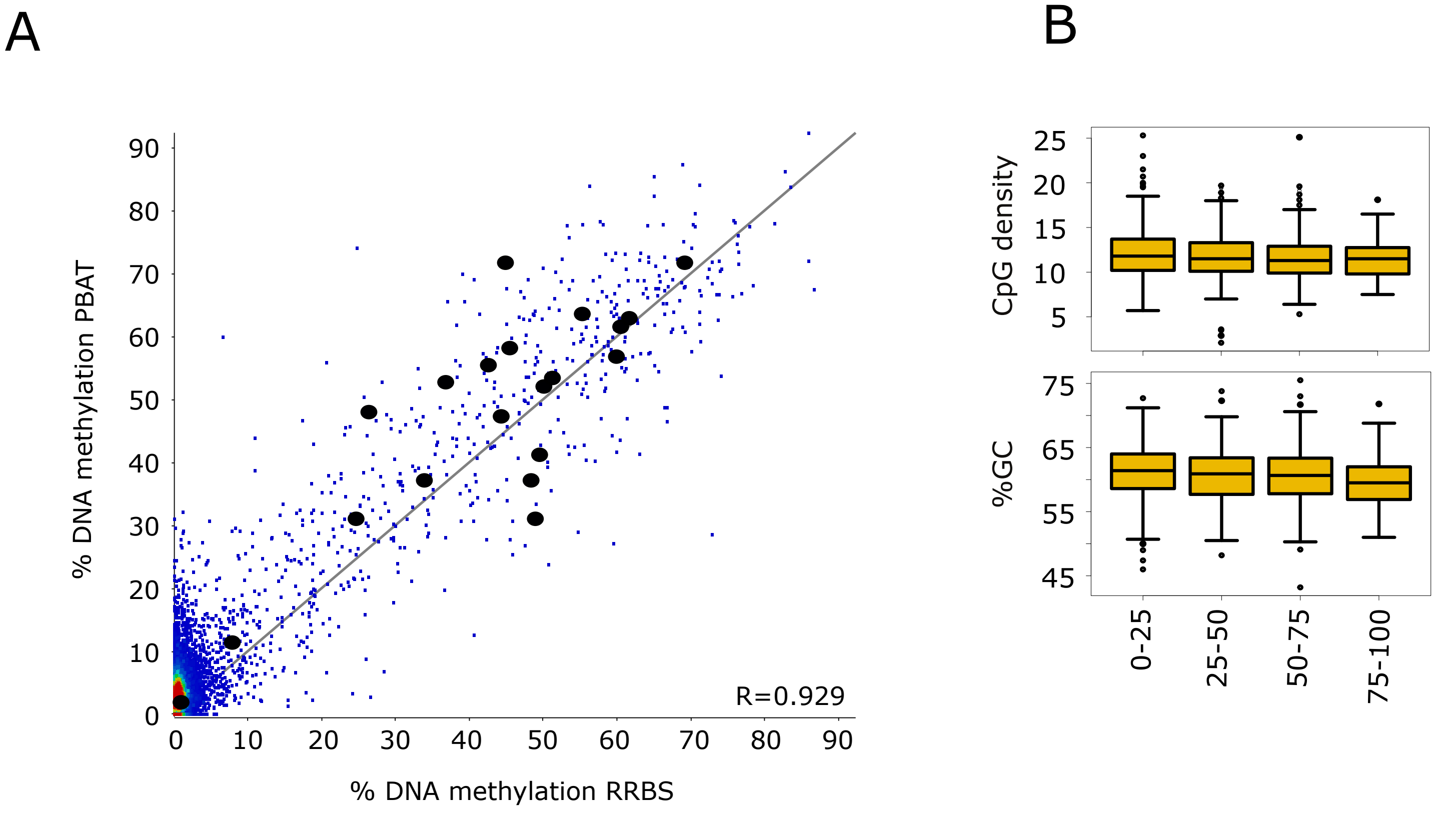

Additional file 3: Figure S2

(A) Scatterplot of CGI methylation in 60-65 μm oocyte RRBS and PBAT datasets. X and Y axis show DNA methylation values in RRBS and PBAT datasets, respectively. R value of 0.929 suggests high level of correlation between these two datasets. Black dots highlight individual gDMRs.

(B) Box whisker plots of CpG density and %GC of CGIs binned by % methylation in 60-65 μm oocytes (PBAT dataset). The numbers of CGIs in each DNA methylation category (from lowest to highest) are: 470, 329, 384 and 63.

Additional file 3: Figure S3

PCA plots for oocyte mRNA-seq libraries.

Top graph represents PCA plot for all RNA-seq datasets from E18.5 to GV oocytes, in which PC1 explains 96.0% of the variance and PC2 a further 2.6%. Bottom graph represents PCA plot with the E18.5 dataset excluded from the analysis, in which PC1 explains 98.4% and PC2 a further 0.9% of the variance.

Figure S3

all RNA-seq datasets (including E18.5 oocytes)

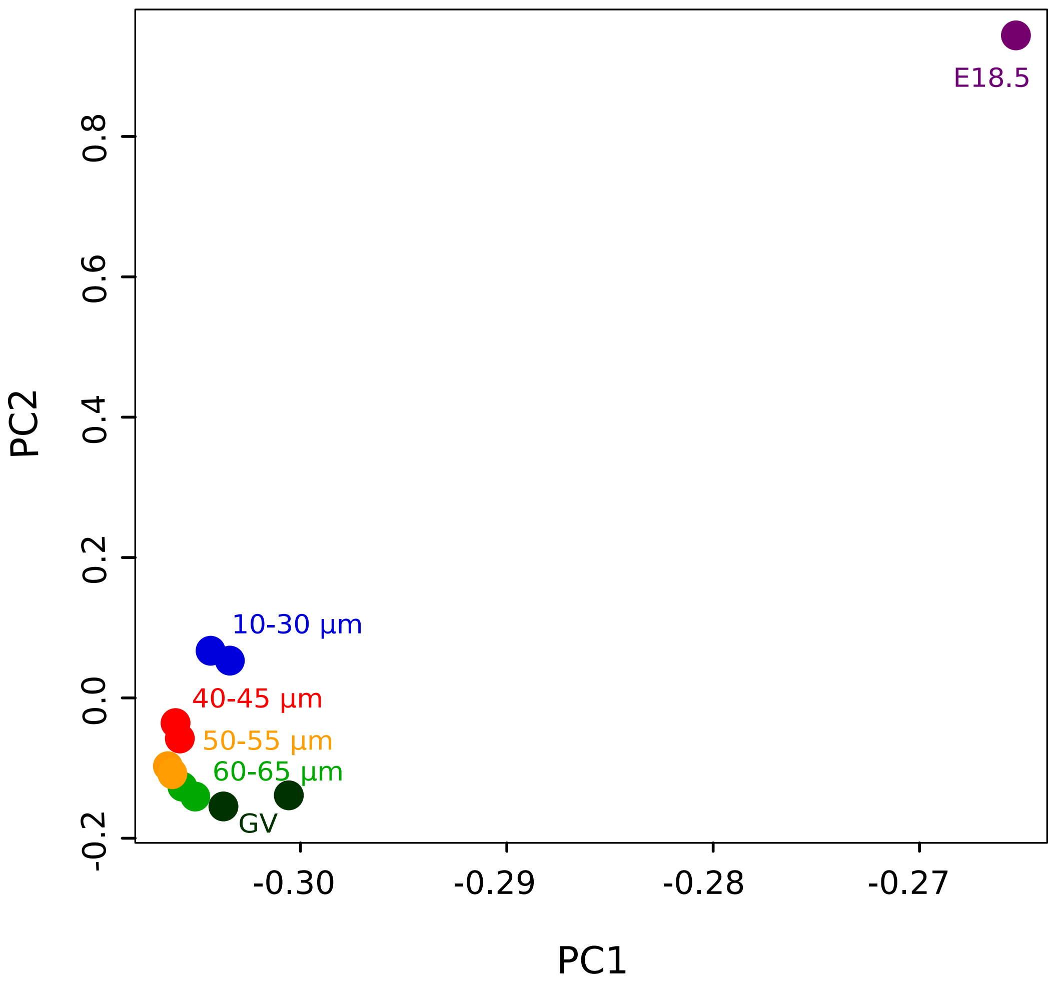

growing oocytes RNA-seq datasets (excluding E18.5 oocytes)

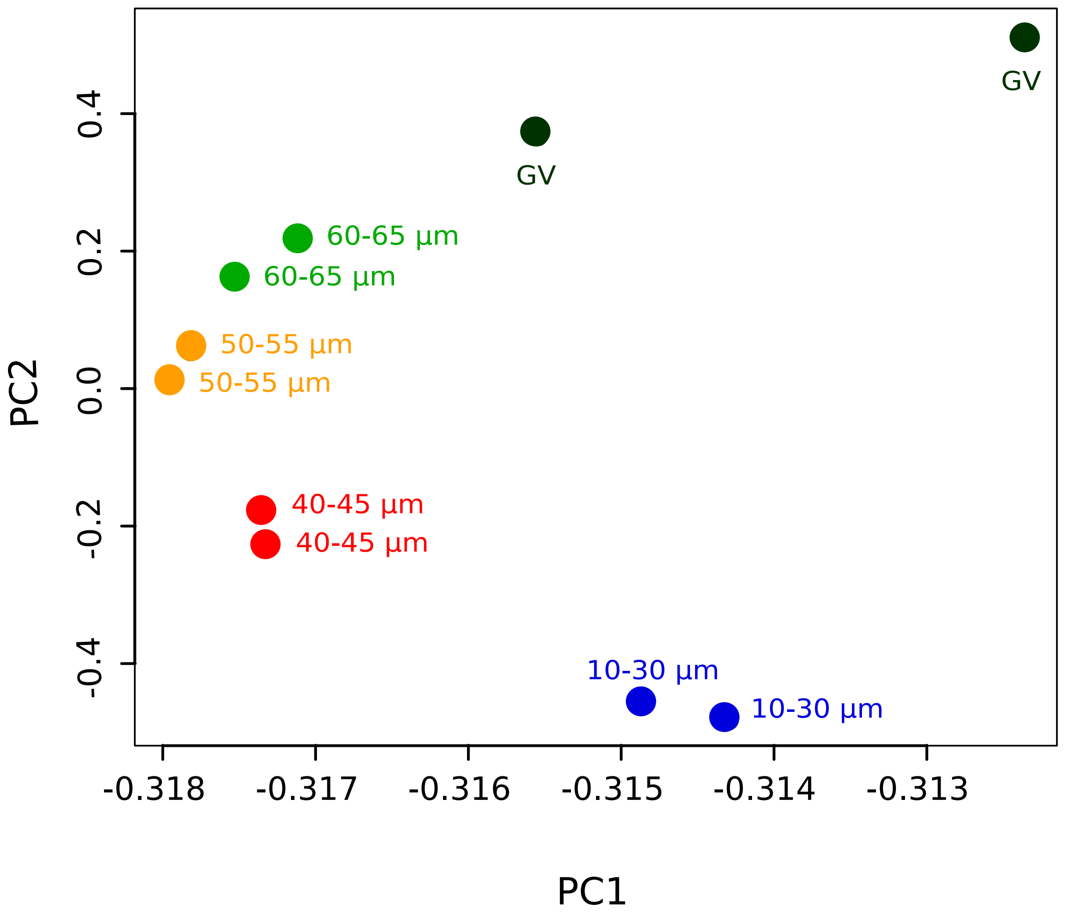

Figure S4

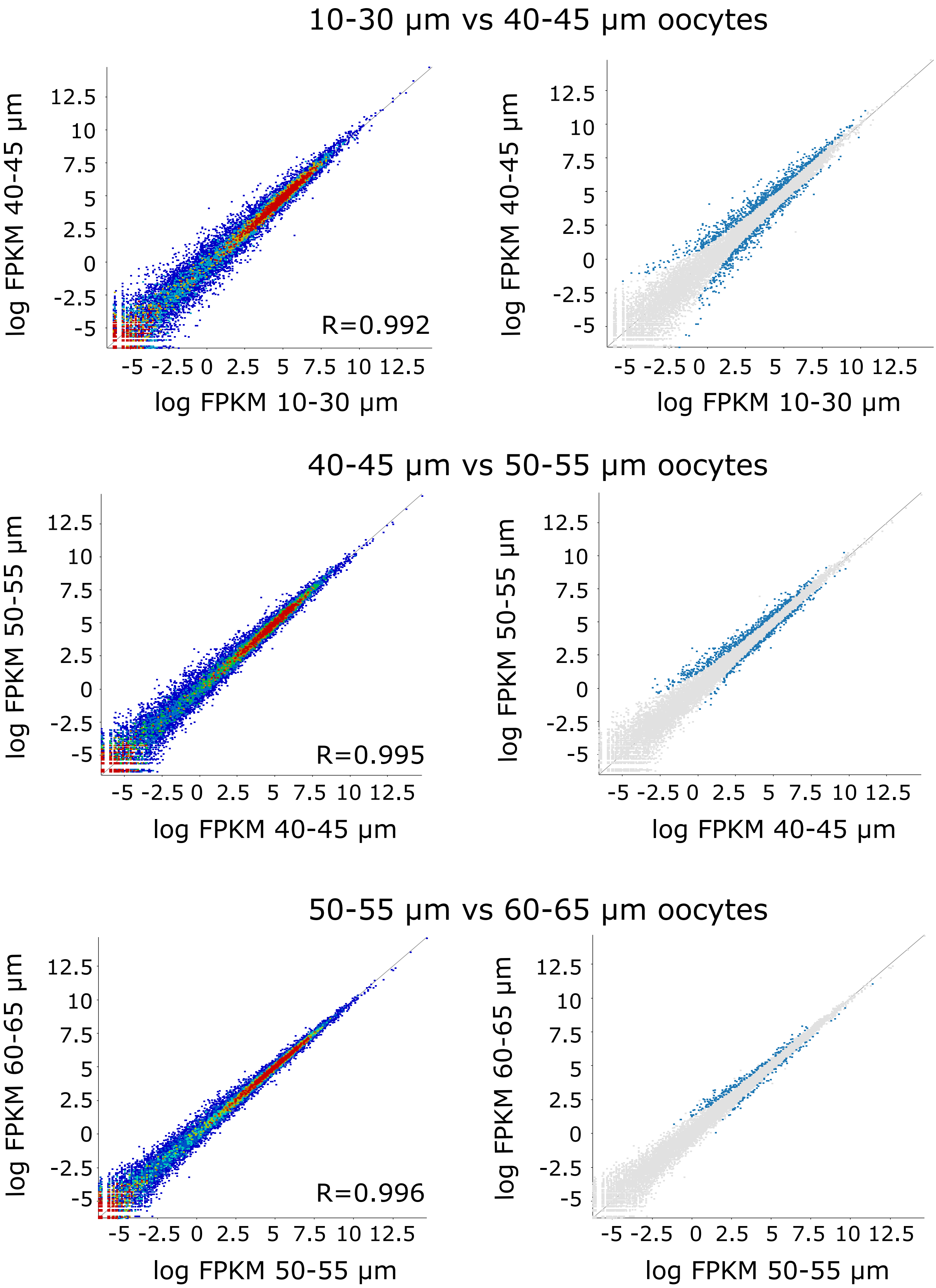

Additional file 3: Figure S4

Scatterplots for pair-wise comparison of RNA-seq datasets from consecutive size populations of growing oocytes. Replicate RNA-seq datasets for each size population were combined for this analysis. The left-hand panels show colour-coding density plots for all 25,965 annotated genes, and right-hand panels highlight differentially expressed genes identified by DESeq2, at a p value ≤0.01. The number of genes identified as differentially expressed in each paired comparison is: 1062 genes between 10-30 µm and 40-45 µm; 717 between 40-45 µm and 50-55 µm; and 278 between 50-55 µm and 60-65 µm.

Figure S5

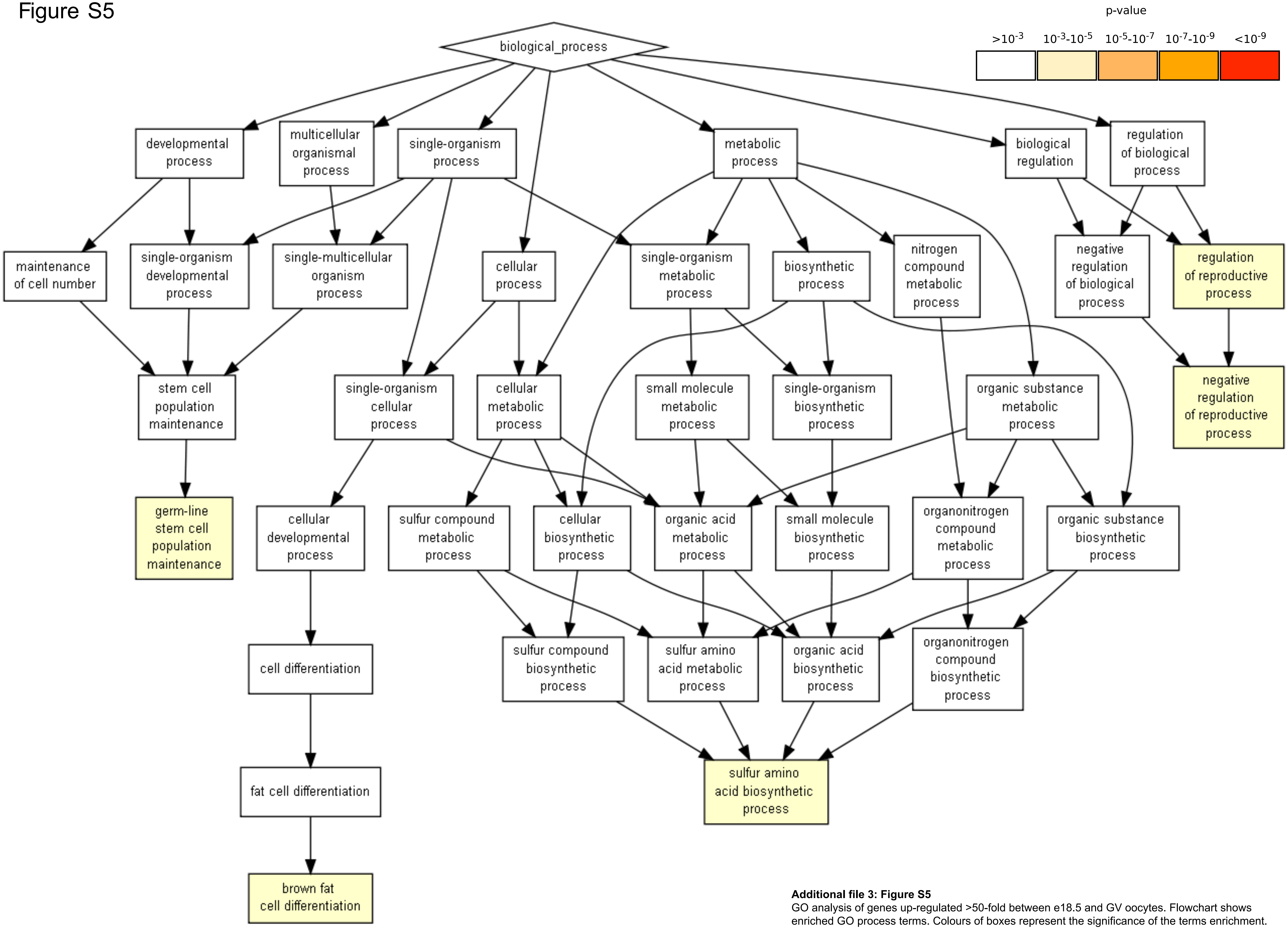

Additional file 3: Figure S5
GO analysis of genes up-regulated >50-fold between e18.5 and GV oocytes. Flowchart shows enriched GO process terms. Colours of boxes represent the significance of the terms enrichment.

Figure S6

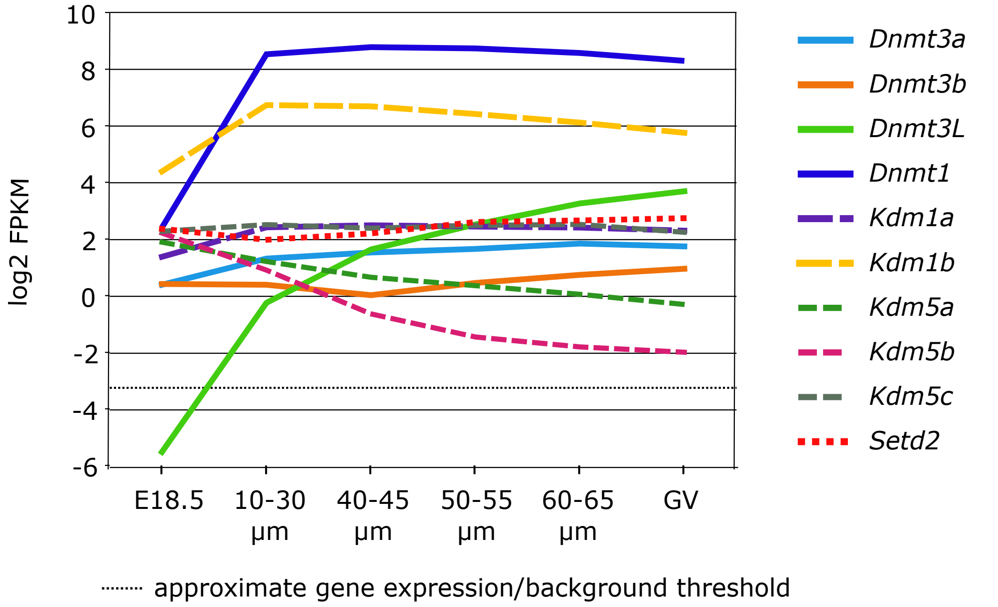

Additional file 3: Figure S6

Graph showing expression levels over oocyte growth of transcripts for Dnmts, Kdm1s and Kdm5s, and SetD2. Dashed line represents an approximate FPKM threshold when genes are considered to be expressed (based on quantification of threshold values for individual datasets.

Figure S7

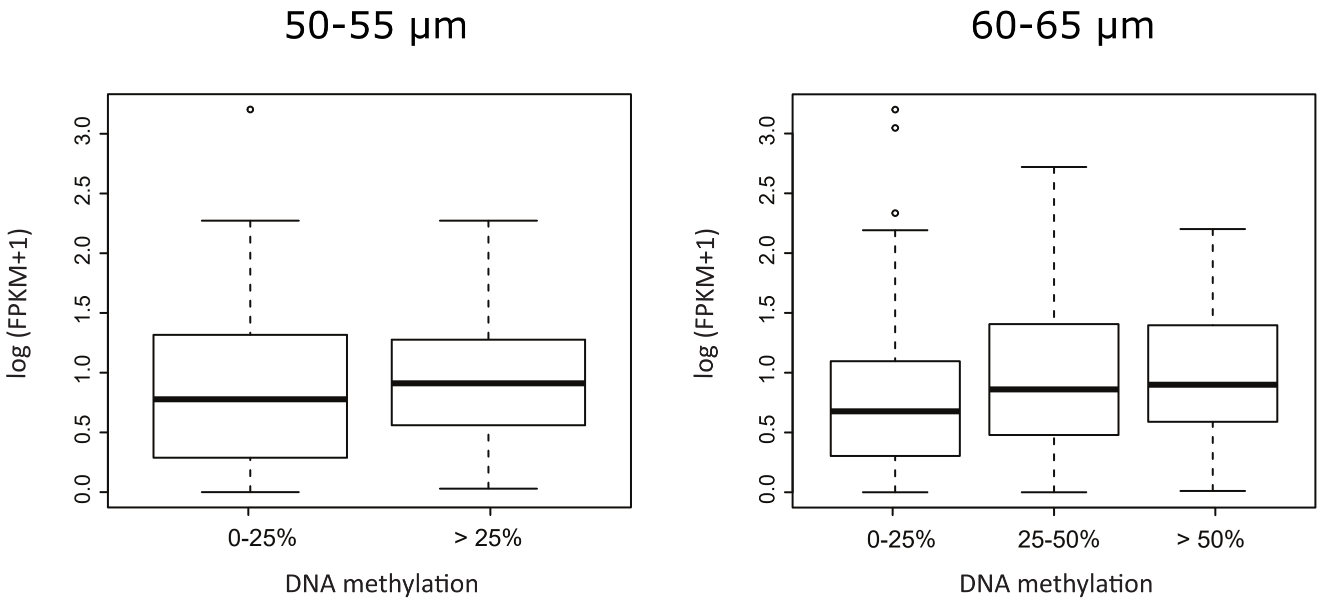

Additional file 3: Figure S7
Methylation level of intragenic CGIs in RRBS datasets in relation to expression level of the corresponding gene. Only CGIs that are methylated in GV oocytes are considered for the analysis. Methylation levels in 50-55 µm and 60-65 µm oocytes are compared with RNA-seq dataset from 50-55 µm and 60-65 µm, respectively. The numbers of CGIs analysed each methylation category (from lowest to highest) are: 335 and 97 for 50-55 µm oocytes and 180, 122 and 130 for 60-65 µm oocytes.

Figure S8

0-25% methylated vs 50-75% methylated CGIs

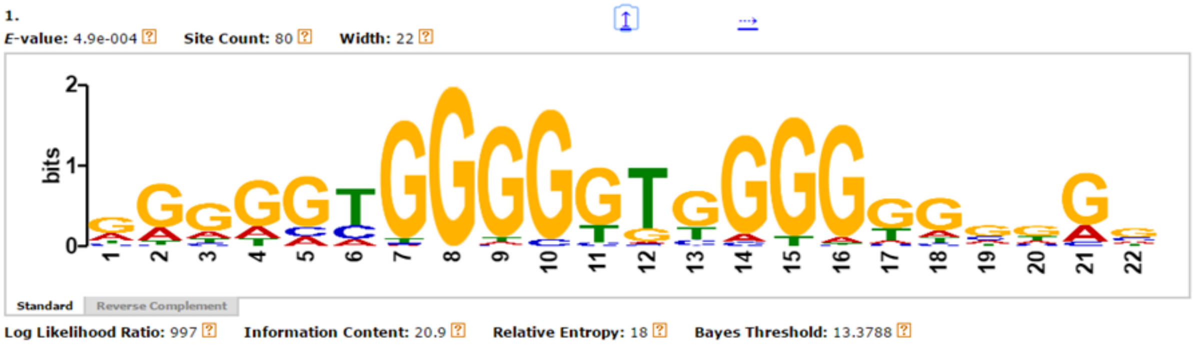

Motif occurence in 0-25% methylated CGIs: 336/470 (71%)
Motif occurence in 50-75% methylated CGIs: 237/384 (62%)

0-25% methylated vs 75-100% methylated CGIs

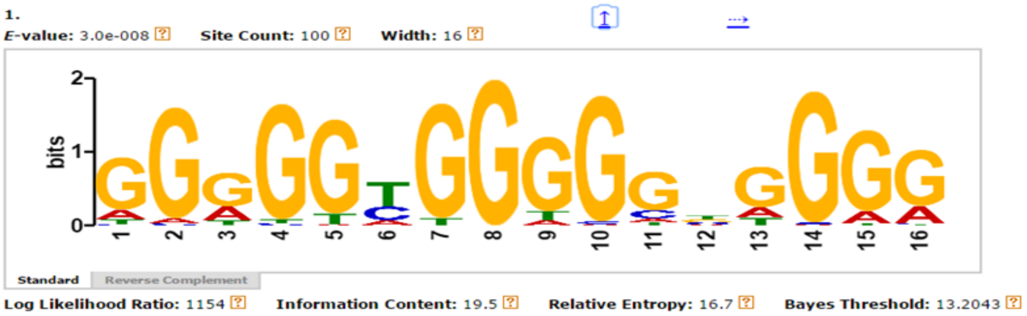

Motif occurence in 0-25% methylated CGIs: 343/470 (73%)
Motif occurence in 75-100% methylated CGIs: 30/63 (48%)

Additional file 3: Figure S8
MEME output of search for motifs enriched in late methylated CGIs (≤25% DNA methylation in 60-65 µm oocytes) compared to CGIs with 50-75% and ≥75% methylation in 60-65 µm oocytes.
